# Roberts syndrome presenting with bilateral Cryptophthalmos: a case report

**DOI:** 10.1093/omcr/omag128

**Published:** 2026-07-12

**Authors:** Muhammad Usman Fareed, Abdur Rehman, Abdullah Shehryar, Shivam Singla, Bhavna Singla, Shabbir Mazari

**Affiliations:** Department of Internal Medicine, Nishtar Medical College and Hospital, Nishtar Road, Gillani Colony, Multan, District Multan, Punjab 60000, Pakistan; Department of Internal Medicine, King Edward Medical University, Neela Gumbad, Anarkali, Lahore, District Lahore, Punjab 54000, Pakistan; Department of Internal Medicine, Allama Iqbal Medical College, Allama Shabbir Ahmed Usmani Road, Quaid-e-Azam Campus, Lahore, District Lahore, Punjab 54550, Pakistan; Department of Internal Medicine, TidalHealth Peninsula Regional, 100 East Carroll Street, Salisbury, Wicomico County, Maryland 21801, United States; Department of Internal Medicine, Erie County Medical Center, 462 Grider Street, Buffalo, Erie County, New York 14215, United States; Department of Internal Medicine, Kabul University of Medical Sciences Abu Ali Ibn Sina, Ata Turk Avenue, Jamal Mena, 3rd District, Kabul 1001, Afghanistan

**Keywords:** Roberts syndrome, ESCO2, cryptophthalmos, cohesinopathy, congenital anomalies

## Abstract

Roberts syndrome (RBS) is a rare autosomal recessive cohesinopathy caused by pathogenic ESCO2 variants and characterized by severe growth restriction, limb reduction defects, and craniofacial anomalies. Cryptophthalmos is classically associated with Fraser syndrome and is not well described in RBS. We report a 7-day-old male neonate born to consanguineous parents who presented with respiratory distress, poor feeding, severe growth restriction, symmetrical limb reduction defects, ambiguous genitalia, and bilateral cryptophthalmos. Prenatal ultrasonography identified limb and genitourinary anomalies. Postnatal evaluation demonstrated multisystem involvement, including horseshoe kidney with hydronephrosis. Molecular testing identified a homozygous pathogenic ESCO2 variant, confirming Roberts syndrome. Although cryptophthalmos initially raised concern for Fraser syndrome, the overall phenotype and genetic findings supported RBS. This case expands the recognized phenotypic spectrum of Roberts syndrome and highlights the importance of molecular testing in complex congenital presentations.

## Introduction

Roberts syndrome (RBS) is a rare autosomal recessive cohesinopathy caused by biallelic pathogenic variants in ESCO2, a gene essential for sister chromatid cohesion during embryogenesis. It classically presents with severe prenatal growth restriction, symmetrical limb reduction defects, and craniofacial anomalies, including cleft lip and palate [1]. A characteristic cytogenetic feature is premature centromere separation with heterochromatin repulsion, producing the distinctive ‘railroad-track’ chromosome appearance. Fewer than 200 cases have been reported worldwide, frequently in consanguineous families, and diagnosis increasingly relies on molecular confirmation through targeted sequencing, multigene panels, or exome-based testing of ESCO2 [2, 3].

In addition to limb and craniofacial abnormalities, RBS may involve ocular, genitourinary, renal, and cardiac systems, reflecting its multisystem developmental impact. Management is multidisciplinary and includes craniofacial and orthopedic interventions, ophthalmologic assessment, renal monitoring, supportive care, and genetic counseling [4]. Reported ocular manifestations have included microphthalmia, hypertelorism, corneal clouding, and other structural abnormalities, although severe eyelid fusion anomalies are exceptionally uncommon.

Cryptophthalmos is a defining feature of Fraser syndrome and has not been clearly documented in RBS, creating diagnostic uncertainty when phenotypic features overlap. Furthermore, blended phenotypes or dual molecular diagnoses have increasingly been recognized in patients with complex congenital anomalies, making careful genetic interpretation essential. We report a neonate with molecularly confirmed Roberts syndrome presenting with bilateral cryptophthalmos, representing a potential novel phenotypic expansion. This case highlights the importance of detailed phenotypic assessment and molecular testing in resolving syndromic ambiguity.

## Case report

A 7-day-old male neonate was admitted with poor feeding, intermittent respiratory distress, limb deformities, and absence of visible ocular structures. He was born at term via spontaneous vaginal delivery to first-degree consanguineous parents after an otherwise uneventful pregnancy without known maternal infections, teratogenic exposures, or significant antenatal medical illness. Antenatal ultrasonography performed at 18 weeks’ gestation had demonstrated bilateral limb reduction defects and suspected genitourinary anomalies. Despite counseling regarding termination, the parents elected to continue the pregnancy. The family history was notable for congenital anomalies in close relatives, raising suspicion of an inherited disorder. There was no documented family history of a previously confirmed syndromic diagnosis.

**Figure 1:**
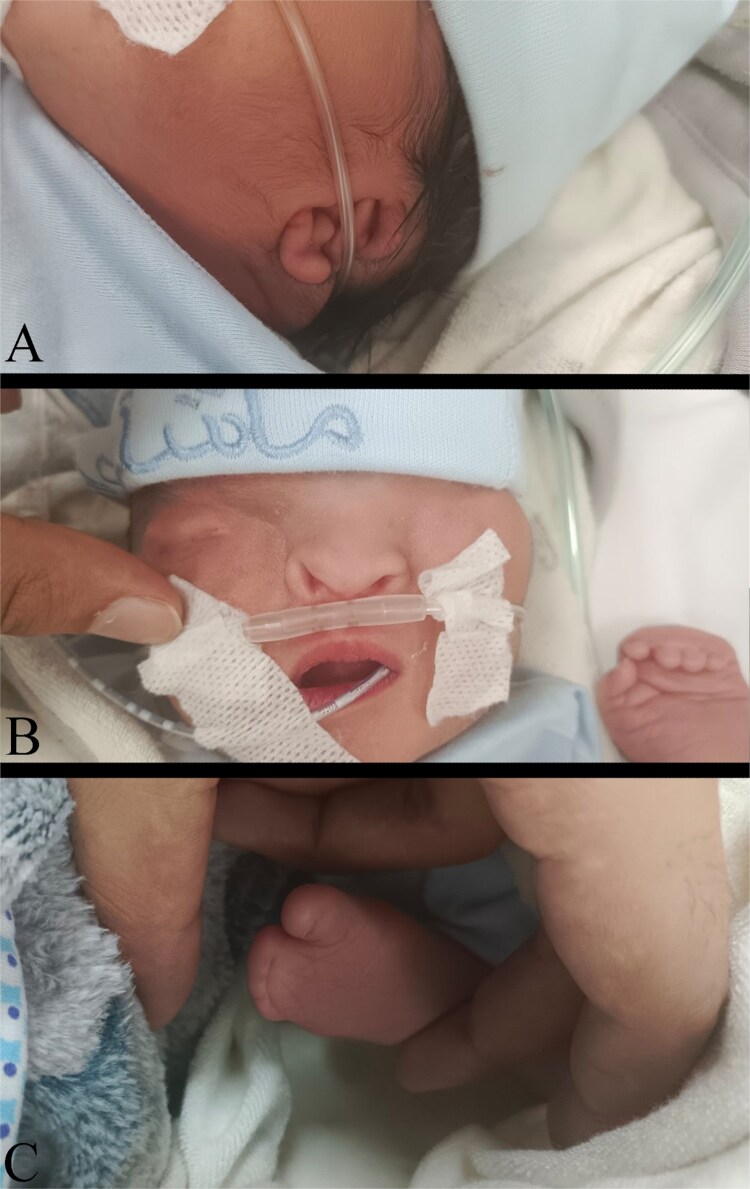
External phenotypic features of the neonate with Roberts syndrome. (A) Lateral facial view showing smooth skin covering the orbital region with absence of identifiable palpebral fissures, consistent with complete cryptophthalmos. (B) Anterior facial view demonstrating bilateral cryptophthalmos with absent eyelid structures and associated nasal deformity; the hand visible adjacent to the face shows marked hypoplasia with shortened digits and syndactyly, consistent with limb reduction defects characteristic of Roberts syndrome. (C) Lower limb view demonstrating severe limb reduction deformities with mesomelic shortening, further supporting the diagnosis of a multisystem congenital disorder.

On admission, the neonate was alert but demonstrated subcostal retractions with intermittent oxygen desaturation. Anthropometric measurements revealed severe growth restriction. Birth weight and current weight were both below the expected range for gestational age. Physical examination showed symmetrical reduction defects of all four limbs with mesomelic shortening and syndactyly. Craniofacial examination revealed micrognathia, a cleft nasal deformity, and complete bilateral cryptophthalmos characterized by smooth skin covering the orbital regions with absence of identifiable palpebral fissures (Fig. 1A–C). No discernible eyelid margins or visible globes were appreciable on external inspection. External genitalia were poorly formed, with a small phallic structure and ambiguous perineum. Cardiovascular examination was unremarkable, while abdominal examination suggested reduced lower abdominal fullness.

Baseline laboratory investigations demonstrated multisystem involvement and are summarized in Table 1. Findings included thrombocytopenia, prolonged coagulation parameters, mild anemia, renal dysfunction, electrolyte imbalance, and elevated inflammatory markers. Urinalysis revealed proteinuria without evidence of urinary tract infection. Septic screening was initiated because of respiratory distress and elevated inflammatory markers, although no definite infectious source was identified clinically.

**Table 1:** Summary of baseline laboratory findings including coagulation profile, hematology, biochemistry, electrolytes, inflammatory markers, and urinalysis.

Parameter	Result	Reference range (Neonate)	Interpretation
Coagulation Profile			
Prothrombin time (patient)	17 sec	≤13 sec	Prolonged
INR	1.5	0.8–1.2	Elevated
APTT	40 sec	≤31 sec	Prolonged
Hematology			
Hemoglobin	13 g/dl	14–20 g/dl	Mild anemia
WBC count	12 500/μl	9000–30 000/μl	Normal
Platelets	104 × 10^9^/l	150–400 × 10^9^/l	Thrombocytopenia
Renal Profile			
Urea	55 mg/dl	10–50 mg/dl	Elevated
Creatinine	1.2 mg/dl	0.5–0.9 mg/dl	Elevated
Liver Function			
Bilirubin (total)	1.5 mg/dl	0.3–1.2 mg/dl	Mildly elevated
ALT	35 U/l	≤40 U/l	Normal
AST	42 U/l	≤40 U/l	Mildly elevated
Electrolytes			
Sodium	132 mmol/l	135–145 mmol/l	Hyponatremia
Potassium	5.6 mmol/l	3.5–5.0 mmol/l	Hyperkalemia
Chloride	100 mmol/l	92–108 mmol/l	Normal
Inflammatory Markers			
ESR	18 mm/hr	0–25 mm/hr	Normal
CRP	12 mg/l	<5 mg/l	Elevated
Urinalysis			
Protein	1+	Negative	Proteinuria
Microscopy	1–2 WBCs, 0–2 RBCs	Within expected limits	No infection

**Figure 2:**
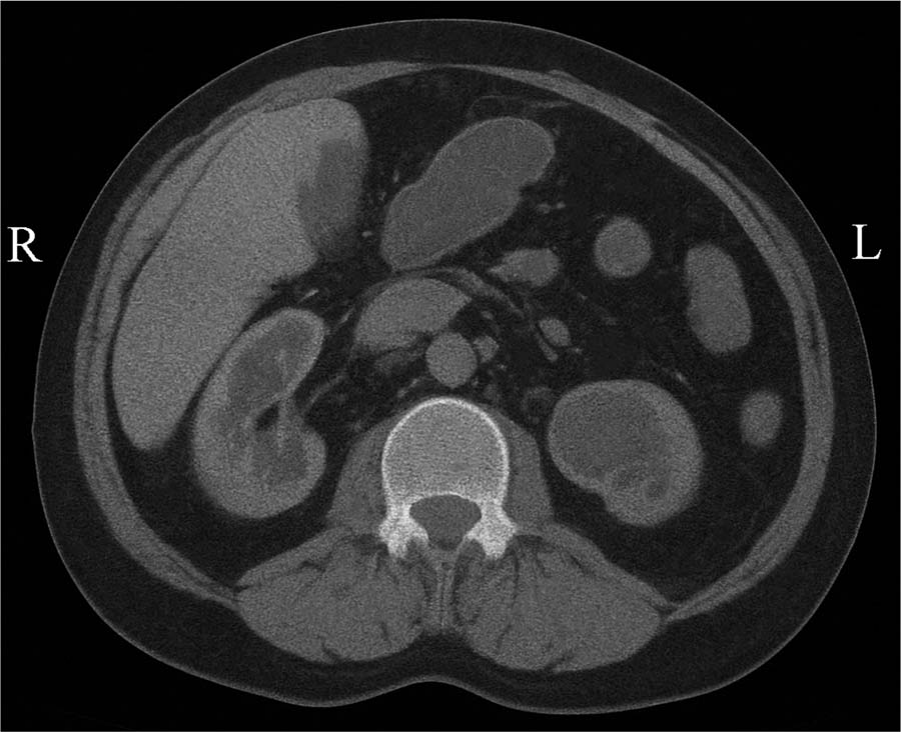
Axial computed tomography image of the abdomen demonstrating renal anomalies.

Radiologic evaluation supported a multisystem congenital disorder. Computed tomography of the abdomen demonstrated fusion of the lower poles of both kidneys forming a horseshoe kidney, with mild bilateral hydronephrosis and a dysplastic bladder, without evidence of hepatic or intestinal pathology (Fig. 2). Skeletal imaging further demonstrated pelvic hypoplasia, consistent with global growth restriction. Dedicated orbital and neuroimaging were not available during the index admission because of resource and logistical constraints.

Given the presence of bilateral cryptophthalmos, Fraser syndrome was initially considered. However, the pattern of symmetrical limb reduction defects, craniofacial anomalies, severe growth restriction, and consanguinity suggested Roberts syndrome as a more likely diagnosis. To clarify the diagnostic uncertainty, next-generation sequencing using a targeted congenital anomaly gene panel was performed through an external reference laboratory and identified a homozygous pathogenic ESCO2 variant, confirming the diagnosis of Roberts syndrome. No additional clearly pathogenic variants associated with common cryptophthalmos-related syndromes were reported within the scope of the assay. However, broader genome-wide testing was not available; therefore, a second independent genetic diagnosis could not be completely excluded.

The neonate was managed with a multidisciplinary approach involving neonatology, ophthalmology, craniofacial surgery, orthopedics, nephrology, and clinical genetics. Initial management focused on airway stabilization, supplemental oxygen, and assisted feeding via orogastric tube to reduce aspiration risk. Long-term planning included staged craniofacial reconstruction, assessment of cryptophthalmos for potential surgical intervention despite poor visual prognosis, orthopedic support for limb abnormalities, and ongoing renal function surveillance. The parents were counseled extensively regarding the diagnosis, prognosis, autosomal recessive inheritance, and recurrence risk. The infant was discharged in a stable condition with close outpatient follow-up and coordinated multidisciplinary care. At early outpatient follow-up, respiratory distress had improved, enteral feeding tolerance was satisfactory with caregiver support, and renal function remained clinically stable, although persistent growth concerns necessitated continued multidisciplinary surveillance.

## Discussion

Roberts syndrome (RBS) is a rare autosomal recessive cohesinopathy characterized by severe prenatal growth failure, limb reduction defects, and craniofacial abnormalities due to biallelic ESCO2 variants. Pathogenic disruption of sister chromatid cohesion produces the classical premature centromere separation (PCS) phenotype, affecting multiple organ systems [5]. While ocular anomalies such as microphthalmia, hypertelorism, or corneal opacities have been reported, cryptophthalmos has not been clearly documented in RBS. This feature is typically associated with Fraser syndrome, caused by mutations in FRAS1, FREM2, GRIP1, or related genes that disrupt epithelial adhesion and eyelid development [6]. The presence of bilateral cryptophthalmos in our patient introduces a diagnostic challenge and suggests broader phenotypic variability in RBS.

The overlap between RBS and Fraser syndrome is clinically significant. Limb anomalies, cleft nasal deformity, severe intrauterine growth restriction, and consanguinity supported RBS in our patient, whereas the ocular defect initially suggested Fraser syndrome. Definitive diagnosis required prenatal imaging, postnatal radiology, and molecular testing, highlighting the necessity of genetic evaluation when classical phenotypes are blurred [7]. In the present case, a homozygous pathogenic ESCO2 variant was identified, a finding biologically consistent with the parental consanguinity reported. Although no additional clearly pathogenic variants were identified within the scope of testing, broader genome-wide sequencing was not available; therefore, a blended phenotype or second independent genetic diagnosis cannot be completely excluded.

Increasing recognition of multilocus disease in medical genetics has demonstrated that some patients with complex congenital anomalies may harbor pathogenic variants in more than one gene, resulting in overlapping or atypical phenotypes. This concept is particularly relevant in cases such as ours, where a hallmark feature of one syndrome appears alongside a molecularly confirmed alternative diagnosis. Accordingly, this case should be interpreted as either an unusual phenotypic expansion of Roberts syndrome or a possible dual-pathology presentation not fully resolved by available testing.

Management underscores challenges in ultra-rare multisystem disorders: airway protection, feeding support, renal surveillance, craniofacial reconstruction, and orthopedic care demand coordinated multidisciplinary involvement. Sparse outcome data complicate evidence-based protocols, and coexisting coagulopathy, renal dysfunction, and complex anatomy may adversely influence prognosis and long-term functional outcomes. Genetic counseling is essential because of autosomal recessive inheritance and the elevated recurrence risk in consanguineous couples.

This case broadens the recognized clinical spectrum of RBS and emphasizes the diagnostic value of molecular testing in syndromic ambiguity. Reporting atypical features refines clinical awareness, improves differential reasoning, and may stimulate investigation into potential cohesin-related effects on craniofacial and ocular morphogenesis. Further cases, expanded sequencing, or registry-level data are needed to determine whether cryptophthalmos represents a rare manifestation of RBS or coincidental dual pathology, underscoring the importance of genomic investigation and interdisciplinary follow-up in complex neonatal anomalies.

## Supplementary Material

Response_to_Reviewers_omag128
