# Supplementary material for: Roberts syndrome presenting with bilateral Cryptophthalmos: a case report
Source: Oxf Med Case Reports. 2026 Jul 12;2026(7):omag128. doi: 10.1093/omcr/omag128 (PMC13356589; doi:10.1093/omcr/omag128)
Supplement: Response_to_Reviewers_omag128 [file response_to_reviewers_omag128.docx]

**Response to Reviewers**
**Manuscript ID:** OMCR-2025-1111
**Title:** *Roberts Syndrome Presenting with Bilateral Cryptophthalmos: A Case Report*

Dear Editor,

We sincerely thank you and the reviewers for the careful evaluation of our manuscript and for the constructive comments provided. We have revised the manuscript extensively in response to all suggestions. Changes have been incorporated into the revised manuscript and highlighted accordingly. We believe these revisions have significantly improved the clarity, scientific rigor, and balance of the report.

Please find our point-by-point responses below.

**Reviewer 1**

**Comment 1:**

Interesting case! Were the mutations homozygous as would be expected with the history of consanguinity?

**Response:**

We thank the reviewer for this insightful comment. We have clarified the genetic findings in the revised manuscript. The identified **ESCO2** pathogenic variant was reported as **homozygous**, which is consistent with the documented history of parental consanguinity. This information has now been added to the Case Presentation and Discussion sections.

**Comment 2:**

What genetic testing was done (single gene, panel, exome, genome)?

**Response:**

We appreciate the reviewer highlighting the need for greater methodological clarity. We have revised the manuscript to specify that **next-generation sequencing using a targeted congenital anomaly gene panel performed through an external reference laboratory** was undertaken. This has now been clearly stated in the Case Presentation.

**Comment 3:**

My concern would be the lack of broad testing potentially missing a second cause for the cryptophthalmos. That can be addressed in the case report.

**Response:**

We agree with the reviewer and appreciate this important point. In the revised manuscript, we have explicitly acknowledged this limitation. We now state that although no additional clearly pathogenic variants were identified within the scope of the performed assay, **broader genome-wide testing was not available; therefore, a second independent genetic diagnosis or blended phenotype cannot be completely excluded**. This statement has been incorporated into both the Case Presentation and Discussion sections.

**Comment 4:**

I am also curious if there was any imaging of the brain and eyes.

**Response:**

We thank the reviewer for this relevant observation. Dedicated orbital and neuroimaging were **not available during the index admission because of resource and logistical constraints**. This limitation has now been added to the Case Presentation section for transparency.

**Reviewer 2**

**Comment 1:**

What genetic testing was done specifically? Was it a single gene, gene panel, exome, or genome?

**Response:**

We appreciate the reviewer’s request for greater specificity. The manuscript has been revised to clarify that **next-generation sequencing using a targeted congenital anomaly gene panel** was performed through an external reference laboratory. This has now been detailed in the Case Presentation.

**Comment 2:**

What is the exact variant? Was it a known pathogenic, or likely pathogenic variant or was it a novel variant?

**Response:**

We thank the reviewer for this important comment. We have clarified in the revised manuscript that the patient was found to harbor a **homozygous pathogenic ESCO2 variant**. In our setting, the externally issued report classified the variant as pathogenic; however, complete annotation details were not fully available in the discharge records. We have therefore reported the confirmed classification transparently while avoiding unsupported overstatement. This clarification has been added to the manuscript.

**Comment 3:**

If it was not a novel variant, please summarize the phenotype reported with this variant.

**Response:**

We appreciate this suggestion. As complete variant-level annotation and prior case linkage data were not available from the external report, we were unable to reliably correlate the specific genotype with previously published phenotype data. We have therefore revised the manuscript to focus on the clinically confirmed diagnosis of Roberts syndrome and have acknowledged this limitation in the Discussion.

**Comment 4:**

How do you know if there is no other genetic condition that might explain the cryptophthalmos? Patients might have two separate conditions and it is well documented in the medical literature.

**Response:**

We fully agree with the reviewer’s concern and appreciate the opportunity to strengthen the manuscript. In response, we have substantially revised the Discussion to acknowledge the possibility of **multilocus disease / dual molecular diagnoses** in patients with complex congenital anomalies. We now explicitly state that, because comprehensive genome-wide testing was unavailable, **a second independent genetic condition contributing to cryptophthalmos cannot be definitively excluded**. We further note that the present case may represent either an unusual phenotypic expansion of Roberts syndrome or a dual-pathology presentation not fully resolved by available testing.

**Additional Revisions Made**

In addition to the reviewer-requested changes, we have also:

- Revised the title to provide a more concise and balanced presentation.
- Shortened and reformatted the Abstract to comply with journal word-count requirements.
- Strengthened the Introduction and Discussion sections.
- Updated the manuscript language for clarity and consistency.
- Ensured formatting compliance with journal submission guidelines.

We are grateful to the reviewers and editorial team for their thoughtful comments, which have substantially improved our manuscript. We hope the revised version is now suitable for reconsideration for publication in *Oxford Medical Case Reports*.

Sincerely,

**Dr. Shabbir Mazari**
Corresponding Author
On behalf of all co-authors
